# Modelling and Mitigating Secondary Crash Risk for Serial Tunnels on Freeway via Lighting-Related Microscopic Traffic Model with Inter-Lane Dependency

**DOI:** 10.3390/ijerph20043066

**Published:** 2023-02-09

**Authors:** Shanchuan Yu, Yu Chen, Lang Song, Zhaoze Xuan, Yi Li

**Affiliations:** 1 National Engineering and Research Center for Mountainous Highways, China Merchants Chongqing Communications Research & Design Institute Co., Ltd., Chongqing 400067, China; 2 School of Smart City, Chongqing Jiaotong University, Chongqing 400067, China; 3 China Everbright Limited Terminus (Shanghai) Information Technology Co., Ltd., Shanghai 200232, China; 4 School of Transportation Science and Engineering, Harbin Institute of Technology, Harbin 150090, China; 5 Logistics Research Center, Shanghai Maritime University, Shanghai 201306, China

**Keywords:** secondary crash, serial tunnels, lighting condition, surrogate safety measure, inter-lane dependency, adaptive tunnel lighting, connected vehicle

## Abstract

This paper models and mitigates the secondary crash (SC) risk for serial tunnels on the freeway which is incurred by traffic turbulence after primary crash (PC) occurrence and location-heterogeneous lighting conditions along serial tunnels. A traffic conflict approach is developed where SC risk is quantified using a surrogate safety measure based on the simulated vehicle trajectories after PC occurs from a lighting-related microscopic traffic model with inter-lane dependency. Numerical examples are presented to validate the model, illustrate SC risk pattern over time, and evaluate the countermeasures for SC, including adaptive tunnel lighting control (ATLC) and advanced speed and lane-changing guidance (ASLG) for connected vehicles (CVs). The results demonstrate that the tail of the stretching queue on the PC occurrence lane, the adjacent lane of the PC-incurred queue, and areas near tunnel portals are high-risk locations. In serial tunnels, creating a good lighting condition for drivers is more effective than advanced warnings in CVs to mitigate SC risk. Combined ATLC and ASLG is promising since ASLG informs CVs of an immediate response to traffic turbulence on the lane where PC occurs and ATLC alleviates SC risks on adjacent lanes via smoothing the lighting condition variations and reducing inter-lane dependency.

## 1. Introduction

**Figure 1:**
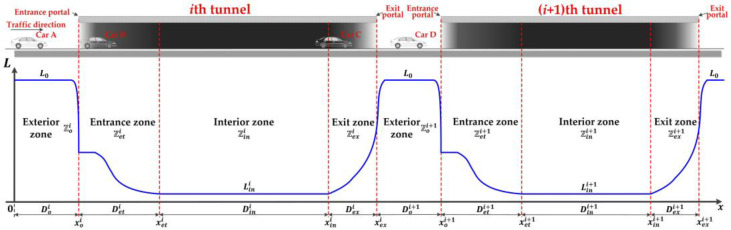
Zone divisions and lighting conditions in serial tunnels.

In the last decade, the mileage of mountainous freeways has rapidly increased in the middle and western regions of China under the national freeway network improvement policy [1]. To improve the road alignment, tunnels are constructed and account for a significant proportion of mountainous freeways. Some of the tunnels are located close to each other and form a section of serial tunnels (successive tunnels, consecutive tunnels, tunnel group, equivalently) [2]. Tunnels are crash-prone in the daytime due to their narrowness with a semi-enclosed structure which leads to low illumination in the interior zone of the tunnel [3,4]. Drivers’ behaviors are strongly influenced by lighting condition variations along serial tunnels, especially by the “black hole” and “white hole” effects near tunnel portals which frequently result in a crash risk [5]. To be specific, sharp lighting difference between the exterior and interior of the tunnel portal reduces the sight distance of the driver in the approaching vehicle, and then the driver experiences visual oscillation incurred by an abrupt lighting transition through the entrance and exit zones of the tunnel [6,7], as represented in Figure 1. In addition, once the crash occurs, a secondary crash (SC) is highly likely to happen since drivers may not have immediate responses to the hazardous traffic turbulence [8]. Therefore, the frequent transitions between daylight and tunnel lighting in serial tunnels require drivers to visually adapt and react quickly, which increases the SC risk and challenges the safety condition of serial tunnels.

SCs are crashes that occur within the spatial and temporal impact range of a primary crash (PC) [9,10,11]. As described above, SC risk in serial tunnels mainly results from traffic turbulence, which is caused by both PC occurrence and lighting variations along serial tunnels. PC-incurred traffic turbulence is a prevalently used contributor [12,13,14,15,16,17,18,19,20]. The impacts of traffic turbulence on SC risk are threefold. The first is the shockwave related to car-following behavior on the lane where PC occurs. The second is the vehicle type heterogeneity since trucks have a lower speed and reduced emergency brake ability in comparison with cars when they respond to the traffic turbulence [21,22,23]. The third is inter-lane dependency for multilane freeways, that vehicles’ speeds are significantly reduced and a shockwave is formed by the queue on the adjacent lane, even though no vehicle in the queue attempts to change the lane [24,25,26]. However, the inter-lane dependency is hardly considered in SC risk modelling. In addition, to the best of our knowledge, there is no literature incorporating location-heterogeneous lighting conditions into the generation of traffic turbulence. Therefore, a lighting-related traffic model with inter-lane dependency is needed to reveal the mechanism of SC risk in serial tunnels.

Accordingly, the countermeasures for SC risk consist of two aspects: immediate informing of traffic turbulence and lighting variations smoothness along serial tunnels. Intelligent transportation systems are constantly being upgraded to make the countermeasures technically feasible. The emergence of connected vehicles (CVs) with advanced speed and lane-changing guidance (ASLG) is considered to be a promising solution for SC mitigation [27,28,29,30], even if its market penetration is at a low level up to date. Adaptive tunnel lighting control (ATLC) smooths the transition between daylight and tunnel lighting [31,32], such that the driver’s visual oscillation with the vehicle’s speed turbulence can be alleviated. However, whether ASLG and ATLC can effectively alleviate SC risk in serial tunnels needs to be further evaluated.

Hence, the aim of this study is to model the SC risk of serial tunnels on the freeway where the impacts of traffic turbulence after PC occurrence and lighting condition variations along serial tunnels on SC risk are described. The countermeasures for SC risk are evaluated, including ATLC for the present and ASLG for CVs in the future mobility.

The remainder of this paper is organized as follows. Section 2 provides a literature review on SC risk modelling and highlights our contribution. The model is formulated in Section 3. In Section 4, scenarios and simulations are conducted. Section 5 provides the results for the model validation, features of SC risks over time, and a countermeasure evaluation for SC risk. Conclusions of this paper and avenues for future research are presented in Section 6. Appendix A demonstrates the calibration of the lighting-related behavioral parameters in the model.

## 2. Literature Review

Compared with PCs which usually result from the driver’s error, SCs are more deterministic and predictable because of their one-way causalities to PCs. The main contributing factors for SC risk include the type and duration of PC, time of day, road geometry, and traffic turbulence upstream of PC, where the last one is presented as the key factor [33].

Two mainstream approaches are developed to link the traffic turbulence with SC risk: crash-based statistical and traffic conflict approaches. In the former, SC risk is modelled as a direct measure, SC occurrence, described by a binary outcome (i.e., crash vs non-crash), according to the records in the crash database [33]. Traditional statistical models and machine learning techniques are developed to link the SC occurrence with traffic turbulence [12,13,14,15,16,17,18,19,20,34,35,36,37,38,39,40,41,42,43,44,45]. Traffic turbulence is quantified by macroscopic traffic flow variables along the spatial impact range of PC, such as average volume, speed, and occupancy, obtained from high-density probe vehicle data or traffic flow sensors with spacing of about 0.5 or 1.0 m on average.

**Figure 2:**
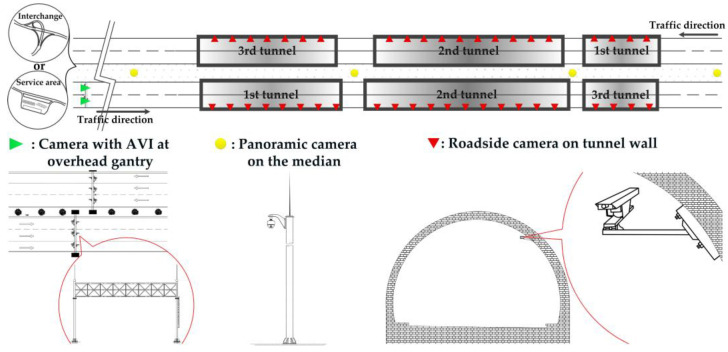
Traffic data availability in section of serial tunnels on Chinese freeway.

However, this method is insufficient for serial tunnels on the Chinese freeway for two main reasons. The first is the unavailability of crash records and the lack of traffic flow sensors along the section. The second is that it cannot describe the traffic turbulence caused by lighting condition variations. The historical crashes are recorded in traffic police centers and are confidential to the public. The traffic-related data are collected from three types of cameras in serial tunnels installed by the freeway management center, as presented in Figure 2. The first is the camera at the overhead gantry a few kilometers upstream the first tunnel. It has the function of automatic vehicle identification (AVI) where the traffic flow variables are available. The second is the panoramic camera on the median of the freeway where a 1 km range can be covered. The third is the roadside camera with 150 m spacing on the tunnel wall which has a relatively narrow side view in the interior of tunnel. The latter two types of cameras merely have the function of incident detection, even though the views of them cover the entire serial tunnels. In other words, when they automatically capture the occurrence of PC, the traffic turbulence after PC occurs cannot be predicted since the real-time traffic flow variables along the serial tunnels are absent.

On the contrary, the traffic conflict approach is suitable for SC risk evaluation in serial tunnels on the Chinese freeway since it was initiated when the data for historical crashes were unavailable or traffic flow conditions were unknown. It can act as a surrogate for safety measures due to the causal relationship between traffic conflict severity and crash occurrence. The traffic conflict severity after PC occurrence is based on simulated vehicle trajectories from a microscopic traffic model and is evaluated by surrogate safety measures (SSMs) [46,47,48,49,50,51]. In microscopic traffic models, car-following (CF) and lane-changing (LC) maneuvers are described on a second or sub-second basis. For example, Li et al. [9] evaluated SC risk in adverse weather where historical crash data were scarce since adverse weather occurs infrequently. The simulated trajectories were obtained from a CF model with limited road surface adhesion and sight distance during adverse weather. The traffic conflict severity was quantified via time-to-collision (TTC), time exposed TTC, and time integrated TTC. Peng and Xu [11] evaluated the effectiveness of ASLG from arrays of roadside variable message signs on SC prevention where the evolutionary traffic condition under roadside ASLG after PC occurs can solely be predicted via simulation. The trajectories were obtained from the microscopic traffic simulator SUMO where CF and LC models. The traffic conflict severity was quantified via TTC and the deceleration rate to avoid the crash (DRAC). Son et al. [30] assessed in-vehicle ASLG in CV environment to prevent SCs. Since the driving behaviors are different in a CV environment, the trajectories were collected from driving simulation experiments and traffic conflict severity was quantified via TTC.

As described above, the key for traffic conflict-based SC analysis is the simulated vehicle trajectories after PC occurrence based on microscopic traffic models. Most CF and LC models are location-homogeneous where the behavioral parameters in the model remain constant during the course of driving [52]. However, they cannot accommodate vehicle maneuvers in serial tunnels where behavioral parameters are location-heterogeneous due to lighting condition variations along the section. In addition, the inter-lane dependency is hardly considered in traffic flow modelling to evaluate SC risk.

In terms of the abovementioned research gaps, the objective of this paper is to develop a traffic conflict approach where the SC risk in serial tunnels is quantified using SSM based on the vehicle trajectories after PC occurrence simulated from a lighting-related microscopic traffic model with inter-lane dependency (LMTID). The main contributions of this study are presented as follows:

- A feasible solution is proposed for SC risk evaluation in serial tunnels of Chinese freeways where there is a lack of traffic flow sensors and an unavailability of crash records.
- The behavioral parameters of CF and LC models are associated with the lighting conditions of different zones in serial tunnels. The link between SC risk in serial tunnels and lighting condition variation is modelled.
- The inter-lane dependency is incorporated into the CF model to describe the impact of traffic turbulence from the PC-occurrence lane on the adjacent lane. The link between SC risk and traffic turbulence from PC is improved for the multilane freeway which conventionally considers the effects of vehicle type heterogeneity and CF-related shockwave on the lane where PC occurs.

## 3. Model Description

The key for traffic conflict-based crash risk analysis in serial tunnels is the simulated vehicle trajectories from a suitable microscopic traffic flow model. The lighting condition variations along the serial tunnels have an impact on drivers’ desired speeds and perceived spacings. To be specific, (1) if the lighting difference (LD) between the exterior and interior of the tunnel portal is sharp, it is hard for the following vehicle to correctly perceive the spacing from preceding vehicles [7]; (2) if the lighting transition (LT) through the portal areas (i.e., entrance and exit zones) is abrupt, the driver has to experience the visual adaptation (VA) with slowing down the vehicle to accommodate the visual oscillation [53,54,55]; (3) the duration of VA reduces as driver travels through serial tunnels since he or she accommodates the consecutive LTs in the portal areas [56]; (4) due to the low illuminance in the interior zone, the driver keeps a relatively low desired speed with carefulness, even though he or she has lower discomfort after VA through the entrance zone [57,58].

Hence, the lighting-related desired speeds and perceived spacings in different locations should be presented by LMTID. A desired measures model is appropriate for the CF part of LMTID, which is one of the mainstream CF models [52]. Intelligent driver model (IDM) is one of the most popular time-continuous desired measures models using desired speed [59]. Due to its broad extrapolation, IDM is extended by following studies from various aspects [60,61,62,63,64,65]. Hence, it is feasible to incorporate the lighting-related desired speed and LD-related perceived spacing into IDM. In addition, the vehicle type heterogeneity and inter-lane dependency in CF can also be described by the IDM-formed model. The former has already been considered to reflect the varieties of speed and braking ability between cars and trucks’ responses to the traffic turbulence [21,22,23]. The latter [24,25,26] has been incorporated into CF models, such as optimal velocity models [66,67], and the similar extension will be used in IDM.

The LC models are divided into two phases, LC decision-making (LCD) and LC process (LCP) modelling. In LCD, rule-based models appear to be the most popular [68,69,70] since they have deterministic rules for the probability of subject vehicle’s LCD. The rule is mainly based on spacings between the subject and surrounding vehicles on multiple lanes. The LCD structure proposed by rule-based models is flexible and CF models can be easily integrated. However, in many of existing LC models, the second phase LCP modelling is not necessary and LC behavior is assumed to be executed instantaneously such that the lateral movement during LC and its effect on traffic are not clearly described. Actually, LCP has a significant impact on the following traffic on both the current and target lanes [71]. In addition, the travel speed during LCP is also responsive to the preceding vehicles on both the current and target lanes. Hence, CF models are used in LCP where the preceding vehicle for the subject vehicle is changeable [72].

It should be noted that behavioral decisions of the drivers might be determined by cultural differences [73,74]. Behavioral parameters, such as comfortable acceleration, CF spacing in a jam situation, the driver’s reaction time, and the minimum necessary spacing are utilized from studies observed in China [23,75]. If LMTID is extended to other countries, the parameters need to be modified via field observations. However, the formulation of LMTID can remain unchanged.

In sum, an IDM-formed model is developed in the CF part of LMTID where lighting-related desired speeds and perceived spacings, vehicle type heterogeneity, and inter-lane dependency are incorporated. The LC part of LMTID consists of a deterministic rule integrated with lighting-related perceived spacing in LCD, and the same CF model of LMTID in LCP.

### 3.1. Definitions, Notations and Assumptions

In a section of serial tunnels with I tunnels, give the ith tunnel with length $D^{i}$. We use the road surface luminance as the approximation for driver visual luminance, as previous studies and current tunnel lighting standards did [76,77]. Let $L\left(x\right)$ denote the road surface luminance (cd/m^2^) with respect to the location x in the daytime where the beginning of the section is at x=0, as demonstrated in Figure 2.

Serial tunnels are divided into four main zones with different characteristics of luminance in the daytime: exterior, entrance, interior, and exit zones, respectively, denoted as ${\mathbb{Z}}_{o}^{i}$, ${\mathbb{Z}}_{et}^{i}$, ${\mathbb{Z}}_{in}^{i}$ and ${\mathbb{Z}}_{ex}^{i}$ for the ith tunnel, respectively. The exterior zone is the open road outside where the exterior environmental luminance $L_{0}$ is constant. $L_{0}$ is the average luminance value focused in a conical aperture of a 20° angle and looking forwards towards a centered point in the tunnel opening [77,78]. The entrance zone is the part of the tunnel where the lighting level is decreasing from the level at the end of the exterior zone to the level of the interior zone in the daytime. The interior zone is the inside of the tunnel when the luminance remains at a constantly low level, $L_{in}^{i}$. The exit zone is the part of the tunnel where the lighting level is increasing from the level at the end of the interior zone to the level of the exterior zone in the daytime. The entrance and exit zones are both referred to as portal areas. The lengths of exterior, entrance, interior and exit zones for the ith tunnel are denoted by $D_{o}^{i}$, $D_{et}^{i}$, $D_{in}^{i}$, and $D_{ex}^{i}$, respectively. The ending locations of exterior, entrance, interior, and exit zones for the ith tunnel are denoted by $x_{o}^{i}$, $x_{et}^{i}$, $x_{in}^{i}$, and $x_{ex}^{i}$, respectively.

In current tunnel lighting standards [76,77], the zone divisions for serial tunnels are more sophisticated. The entrance zone is further divided into two subzones: the threshold and transition zones. The exterior zone is classified into three types: access, connecting, and parting zones. However, the simplified zone divisions in our study will not change the formulation of LMTID and can improve the readability of the paper.

The CF part of LMTID is developed via IDM. The lighting-related component for CF considers the desired speed of subject vehicle affected by LT in the portal area and low illuminance in the interior zone, and the perceived spacing of following vehicle affected by LD between the exterior and interior of the tunnel portal. As a result, the following definitions and assumptions are presented.

**Definition** **1.** *The desired speed of the subject car at a location is the average speed at the same location when no surrounding car appears*.

**Definition** **2.** *LD for the subject vehicle is the ratio of luminance between the location of the preceding vehicle and location of the subject vehicle at the same time*.

**Definition** **3.** *LT for the subject vehicle is the ratio of luminance between the present location of the subject vehicle and where the vehicle was 0.2 s ago*.

**Assumption** **1.** *The perceived distance of the driver in the subject vehicle is determined by LD. If the spacing between the subject vehicle and the preceding vehicle is smaller than the perceived distance of the driver in the subject vehicle, the location and speed of the preceding vehicle can be correctly recognized by the subject vehicle. Otherwise, the preceding vehicle “disappears” in the vision of the driver in the subject vehicle, the spacing and speed difference perceived as maximum perceived distance and zero, respectively*.

**Assumption** **2.** *In the portal areas, whether the driver’s VA happens is determined by LT. The VA duration and the desired speed reduction during VA are derived from LT, independently. If VA does not happen in the entrance zone, the desired speed linearly changes between the end of the exterior zone and the beginning of interior zone. If VA does not happen in the exit zone, the desired speed linearly changes between the end of interior zone and the beginning of exterior zone*.

**Assumption** **3.** *In serial tunnels, driver’s VA duration is reduced by the number of times that VAs have been experienced*.

Other assumptions in IDM are involved in the CF submodel of LMTID. Let $x_{n}\left(t\right)$, $v_{n}\left(t\right)$, and $a_{n}\left(t\right)$ denote the location, speed, and acceleration of the vehicle n at time t, respectively. For the vehicle n, the preceding vehicles on the same and adjacent lanes are denoted by the subscripts $\left(n-1\right)$ and $\left(k-1\right)$, respectively. The following vehicles on the same and adjacent lanes are denoted by the subscripts $\left(n+1\right)$ and k, respectively. 

$\Delta x_{n}^{1}\left(t\right)$ ($\Delta x_{n}^{2}\left(t\right)$) and $s_{n}^{1}\left(t\right)$ ($s_{n}^{2}\left(t\right)$) denote the spacing from the preceding vehicles on the same (adjacent) lane to the vehicle n and the spacing from the front edge of the vehicle n to the rear end of the preceding vehicles on the same (adjacent) lane at time t, respectively. We have $\Delta x_{n}^{1}\left(t\right)=x_{n-1}\left(t\right)-x_{n}\left(t\right)$, $s_{n}^{1}\left(t\right)=\Delta x_{n}^{1}\left(t\right)-l_{n-1}$ where $l_{n-1}$ denotes the length of the vehicle $\left(n-1\right)$, and $\Delta x_{n}^{2}\left(t\right)=x_{k-1}\left(t\right)-x_{n}\left(t\right)$, $s_{n}^{2}\left(t\right)=\Delta x_{n}^{2}\left(t\right)-l_{k-1}$ where $l_{k-1}$ denotes the length of the vehicle $\left(k-1\right)$. For the vehicle k, we have $\Delta x_{k}^{1}\left(t\right)=x_{k-1}\left(t\right)-x_{k}\left(t\right)$, $s_{k}^{1}\left(t\right)=\Delta x_{k}^{1}\left(t\right)-l_{k-1}$ and $\Delta x_{k}^{2}\left(t\right)=x_{n}\left(t\right)-x_{k}\left(t\right)$, $s_{k}^{2}\left(t\right)=\Delta x_{k}^{2}\left(t\right)-l_{n}$ where $l_{n}$ denotes the length of the vehicle n. The length varies according to vehicle type. 

Let $\Delta v_{n}^{1}\left(t\right)$ ($\Delta v_{n}^{2}\left(t\right)$) denote the speed difference between the vehicle n and the preceding vehicle on the same (adjacent) lane, i.e., $\Delta v_{n}^{1}\left(t\right)=v_{n-1}\left(t\right)-v_{n}\left(t\right)$ and $\Delta v_{n}^{2}\left(t\right)=v_{k-1}\left(t\right)-v_{n}\left(t\right)$. 

$L_{d}^{1}\left(x_{n}\left(t\right)\right)$ ($L_{d}^{2}\left(x_{n}\left(t\right)\right)$) denotes LD on the same (adjacent) lane at time t and we have $L_{d}^{1}\left(x_{n}\left(t\right)\right)=\frac{L\left(x_{n-1}\left(t\right)\right)}{L\left(x_{n}\left(t\right)\right)}$ ($L_{d}^{2}\left(x_{n}\left(t\right)\right)=\frac{L\left(x_{k-1}\left(t\right)\right)}{L\left(x_{n}\left(t\right)\right)}$) according to Definition 2.$\;L_{t}\left(x_{n}\left(t\right)\right)$ denotes LT at time t and we have $L_{t}\left(x_{n}\left(t\right)\right)=\frac{L\left(x_{n}\left(t\right)\right)}{L\left(x_{n}\left(t-\Delta t\right)\right)}$ where Δt=0.2 s according to Definition 3.

### 3.2. Formulation of LMTID

#### 3.2.1. Lighting-Related Behavioral Parameters

Since the derivations of behavioral parameters in CF and LC models are similar, we introduce them in the CF scenario. When the driver accesses the tunnel portals, his or her perceived distance for the preceding vehicle is affected by LD. Let $d_{n}^{1}\left(\cdot \right)$ denote the perceived distance of the driver in the subject vehicle for the preceding vehicle on the same lane (m), derived from $L_{d}^{1}\left(x_{n}\left(t\right)\right)$. The LD-related $d_{n}^{1}\left(\cdot \right)$ has been investigated by previous studies via a gray target recognition experiment [7], as presented in Equation (1).

(1) $$d_{n}^{1}\left(L_{d}^{1}\left(x_{n}\left(t\right)\right)\right)=\left\{\begin{array}{cc} \frac{172}{1+\exp\left(-76.8L_{d}^{1}\left(x_{n}\left(t\right)\right)+3.4\right)}+25.2\left(m\right) & \mathrm{if}\;L\left(x_{n-1}\left(t\right)\right)\leq L\left(x_{n}\left(t\right)\right)\\ \frac{198}{1+\exp\left(-70.4L_{d}^{1}\left(x_{n}\left(t\right)\right)+2.3\right)}+3.02\left(m\right) & \mathrm{if}\;L\left(x_{n-1}\left(t\right)\right)>L\left(x_{n}\left(t\right)\right) \end{array}\right.$$

In Equation (1), the first and second equations show the perceived distances of drivers accessing entrance and exit portals, respectively. Let ${\hat{s}}_{n}^{1}\left(t\right)$ and $\Delta {\hat{v}}_{n}^{1}\left(t\right)$ denote the perceived spacing and speed difference on the same lane, respectively. In terms of Assumption 1, we have

(2) $${\hat{s}}_{n}^{1}\left(t\right)=\left\{\begin{array}{l} s_{\max}\;\;\;\;\;\mathrm{if}\;s_{n}^{1}\left(t\right)>d_{n}^{1}\left(L_{d}^{1}\left(x_{n}\left(t\right)\right)\right)\\ s_{n}^{1}\left(t\right)\;\;\;\;\;\mathrm{if}\;s_{n}^{1}\left(t\right)\leq d_{n}^{1}\left(L_{d}^{1}\left(x_{n}\left(t\right)\right)\right) \end{array}\right.$$

(3) $$\Delta {\hat{v}}_{n}^{1}\left(t\right)=\left\{\begin{array}{cc} 0 & \mathrm{if}\;s_{n}^{1}\left(t\right)>d_{n}^{1}\left(L_{d}^{1}\left(x_{n}\left(t\right)\right)\right)\\ \Delta v_{n}^{1}\left(t\right) & \mathrm{if}\;s_{n}^{1}\left(t\right)\leq d_{n}^{1}\left(L_{d}^{1}\left(x_{n}\left(t\right)\right)\right) \end{array}\right.$$

where $s_{\max}$ is the maximum perceived spacing of drivers (m).

When the driver travels through the portal areas, whether he or she experiences VA is affected by LT. Let $T_{a}\left(\cdot \right)$ and $b_{a}\left(\cdot \right)$ denote the VA duration (s) and the ratio of desired speed reduction during VA, respectively. Let $k_{1}$ and $k_{2}$ denote the thresholds for VA occurrences at entrance and exit zones, respectively. The LT-related $T_{a}\left(\cdot \right)$ and $b_{a}\left(\cdot \right)$ has been investigated by our field observation described in Appendix A, as presented in Equations (4) and (5):

(4) $$T_{a}\left(L_{t}\left(x_{n}\left(t\right)\right)\right)=\left\{\begin{array}{ll} -2.976\ln L_{t}\left(x_{n}\left(t\right)\right)-11.188\left(s\right) & L_{t}\left(x_{n}\left(t\right)\right)\leq k_{1}\\ 1.275\ln L_{t}\left(x_{n}\left(t\right)\right)-5.470\left(s\right) & L_{t}\left(x_{n}\left(t\right)\right)\geq k_{2} \end{array}\right.$$

(5) $$b_{a}\left(L_{t}\left(x_{n}\left(t\right)\right)\right)=\left\{\begin{array}{ll} 41.841L_{t}\left(x_{n}\left(t\right)\right)-0.059 & L_{t}\left(x_{n}\left(t\right)\right)\leq k_{1}\\ -0.001L_{t}\left(x_{n}\left(t\right)\right)+1.100 & L_{t}\left(x_{n}\left(t\right)\right)\geq k_{2} \end{array}\right.$$

where $k_{1}=0.025$ and $k_{2}=73$, of which the derivations are described in Appendix A. 

When the driver traverses the serial tunnels, his or her VA duration is reduced by the number of times that VAs have been experienced according to Assumption 3. Let $\delta \left(\xi \right)$ denote the reduction coefficient on the VA duration where ξ denotes the number of times that VAs have been experienced. As the experimental results in Cui et al. [56] indicate, $\delta \left(\xi \right)$ is presented as the following.

(6) $$\delta \left(\xi \right)=\left\{\begin{array}{ll} -0.242\ln\xi +0.99 & 1\leq \xi \leq 5\\ 0.6 & \xi >5 \end{array}\right.$$

Due to the constantly low illuminance in the interior zone, the driver keeps a constantly lower desired speed, even though he or she has lower discomfort after VA through the entrance zone. Let $b_{in}\left(\cdot \right)$ denotes the ratio of the desired speed reduction in the interior zone with respect to the luminance $L_{in}^{i}$. The lighting-related $b_{in}\left(\cdot \right)$ has been investigated by our field observation described in Appendix A, as presented in Equation (7).

(7) $$b_{in}\left(L_{in}^{i}\right)=1.818\times 2^{0.157\log L_{in}^{i}}-0.944$$

In sum, the desired speeds $\tilde{V}\left(\cdot \right)$ for zones along serial tunnels are presented as follows. For exterior and interior zones, we have

(8) $$\tilde{V}\left(x_{n}\left(t\right)\right)=\left\{\begin{array}{ll} v_{n}^{0} & x_{n}\left(t\right)\in {\mathbb{Z}}_{o}^{i},i=1,\ldots ,I\\ b_{in}\left(L_{in}^{i}\right)v_{n}^{0} & x_{n}\left(t\right)\in {\mathbb{Z}}_{in}^{i},i=1,\ldots ,I \end{array}\right.$$

where $v_{n}^{0}$ denotes the desired speed on the open road for the vehicle n. For portal areas, in terms of Assumption 2, if $k_{1}<L_{t}\left(x_{n}\left(t\right)\right)<k_{2}$, the VA will not happen and we have

(9) $$\tilde{V}\left(x_{n}\left(t\right)\right)=\left\{\begin{array}{ll} \left[\frac{1-b_{in}\left(L_{in}^{i}\right)}{x_{o}^{i}-x_{et}^{i}}\left(x_{n}\left(t\right)-x_{o}^{i}\right)+1\right]v_{n}^{0} & x_{n}\left(t\right)\in {\mathbb{Z}}_{et}^{i},i=1,\ldots ,I\\ \left[\frac{b_{in}\left(L_{in}^{i}\right)-1}{x_{in}^{i}-x_{ex}^{i}}\left(x_{n}\left(t\right)-x_{ex}^{i}\right)+1\right]v_{n}^{0} & x_{n}\left(t\right)\in {\mathbb{Z}}_{ex}^{i},i=1,\ldots ,I \end{array}\right.$$

If $L_{t}\left(x_{n}\left(t\right)\right)\leq k_{1}$ or $L_{t}\left(x_{n}\left(t\right)\right)\geq k_{2}$, the VA will happen and we have

(10) $$\tilde{V}\left(x_{n}\left(\tau \right)\right)=b_{a}\left(L_{t}\left(x_{n}\left(t\right)\right)\right)v_{n}\left(t\right),\;\tau \in \left[t,t+\delta \left(\xi \right)T_{a}\left(L_{t}\left(x_{n}\left(t\right)\right)\right)\right]$$

#### 3.2.2. CF Part of LMTID

Based on IDM, the dynamical equation of the vehicle n in the CF submodel of LMTID is given by

(11) $$a_{n}\left(t\right)=a_{n}^{\max}\left[1-{\left(\frac{v_{n}\left(t\right)}{\tilde{V}\left(x_{n}\left(t\right)\right)}\right)}^{\beta }-{\left(\frac{{\tilde{s}}_{n}^{1}\left(t\right)}{{\hat{s}}_{n}^{1}\left(t\right)}\right)}^{2}\right]$$

(12) $${\tilde{s}}_{n}^{1}\left(t\right)=s_{n}^{\mathrm{jam}}+v_{n}\left(t\right){\tilde{h}}_{n}-\frac{v_{n}\left(t\right)\min\left(\Delta {\hat{v}}_{n}^{1}\left(t\right),\lambda \Delta {\hat{v}}_{n}^{2}\left(t\right)\right)}{2\sqrt{a_{n}^{\max}a_{n}^{\mathrm{comf}}}}$$

where $a_{n}^{\max}$ and $a_{n}^{\mathrm{comf}}$ denote the maximum acceleration and comfortable deceleration, respectively. $s_{n}^{\mathrm{jam}}$ denotes the minimum spacing at the standstill situation. ${\tilde{h}}_{n}$ is the time gap. λ is the response coefficient of the relative speed between vehicles on the subject and adjacent lanes.

In the model, $\tilde{V}\left(x_{n}\left(t\right)\right)$ in Equation (11) presents the impact of location-heterogeneous lighting conditions along serial tunnels. The impact of traffic turbulence is reflected from three aspects. First, the model can simulate the shockwave after a given PC occurs in a recursive way. Second, the inter-lane dependency is described by the last term of Equation (12). Third, the different responses to traffic due to the vehicle type heterogeneity are presented by different behavioral parameters. For four types of vehicle-following patterns, desired speeds on the open road, minimum spacings at standstill situations, maximum accelerations, and comfortable accelerations are heterogeneous.

In terms of the dynamical equation, the key in the CF submodel of LMTID is the acceleration, $a_{n}\left(t\right)$, determined by five variables, $v_{n}\left(t\right)$, $x_{n}\left(t\right)$, ${\tilde{s}}_{n}^{1}\left(t\right)$, $\Delta {\hat{v}}_{n}^{1}\left(t\right)$, and $\Delta {\hat{v}}_{n}^{2}\left(t\right)$. We denote acceleration as $a_{n}\left(v_{n}\left(t\right),x_{n}\left(t\right),{\hat{s}}_{n}^{1}\left(t\right),\Delta {\hat{v}}_{n}^{1}\left(t\right),\Delta {\hat{v}}_{n}^{2}\left(t\right)\right)$ for simplification.

#### 3.2.3. LC Part of LMTID

The LC submodel of LMTID is divided into two phases, Lv et al.’s [75] incentive-based rule integrated with LD-related perceived spacing in LCD, and the same model of the CF submodel of LMTID in LCP. It should be noted that inter-lane dependency only exists in CF behavior while the queue forms on adjacent lane. Therefore, λ=0 for LCP.

**Figure 3:**
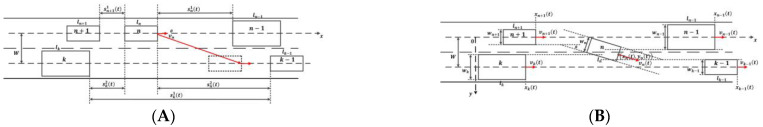
The process and risk for lane-changing on freeways. (**A**) LCD and LCP; (**B**) crash risk during LCP.

Incentive-based models are the variants of Gipps-type rule-based models which are governed by two LC rules, i.e., incentive and security criterion [79,80]. The idea behind the incentive-based models is intuitive and straightforward where there is only one “advantage” value, compared against a threshold value for final decision making. The subject and surrounding vehicles are demonstrated in Figure 3A. The rule in LCD is symmetrical and presented as follows.

Vehicle n can change lane with a probability $p_{1}$ if

(13) $${\hat{s}}_{n}^{2}\left(t\right)>{\hat{s}}_{n}^{1}\left(t\right),\;{\hat{s}}_{n}^{2}\left(t\right)>s_{\min},\;\Delta {\hat{v}}_{n}^{2}\left(t\right)\geq \Delta {\hat{v}}_{n}^{1}\left(t\right),\;s_{k}^{2}\left(t\right)>s_{\mathrm{safe}}$$

Vehicle n can change lane with a probability $p_{2}$ if

(14) $${\hat{s}}_{n}^{2}\left(t\right)>{\hat{s}}_{n}^{1}\left(t\right),\;{\hat{s}}_{n}^{2}\left(t\right)>s_{\min},\;\Delta {\hat{v}}_{n}^{2}\left(t\right)<\Delta {\hat{v}}_{n}^{1}\left(t\right),\;s_{k}^{2}\left(t\right)>s_{\mathrm{safe}}$$

Vehicle n can change lane with a probability $p_{3}$ if

(15) $${\hat{s}}_{n}^{2}\left(t\right)\leq {\hat{s}}_{n}^{1}\left(t\right),\;{\hat{s}}_{n}^{2}\left(t\right)>s_{\min},\;\Delta {\hat{v}}_{n}^{2}\left(t\right)\geq \Delta {\hat{v}}_{n}^{1}\left(t\right),\;s_{k}^{2}\left(t\right)>s_{\mathrm{safe}}$$

Vehicle n does not change lane, otherwise.

where $p_{1}>p_{2}\geq p_{3}$. ${\hat{s}}_{n}^{1}\left(t\right)$, ${\hat{s}}_{n}^{2}\left(t\right)$, $\Delta {\hat{v}}_{n}^{1}\left(t\right)$, $\Delta {\hat{v}}_{n}^{2}\left(t\right)$ are the perceived values for $s_{n}^{1}\left(t\right)$, $s_{n}^{2}\left(t\right)$, $\Delta v_{n}^{1}\left(t\right)$ and $\Delta v_{n}^{2}\left(t\right)$, respectively, of which the definitions are similar to Equations (2) and (3). $s_{\mathrm{safe}}$ is the safe distance for lane changing where $s_{\mathrm{safe}}=\max\left\{s_{\min},s_{k}^{b}\right\}$. $s_{\min}$ denotes the minimum necessary distance for the change.$\;s_{k}^{b}$ denotes the braking distance of the following vehicle on the target lane (i.e., vehicle k in Figure 3A), as presented in Equation (16):

(16) $$s_{k}^{b}=v_{k}\left(t\right)t_{k}^{r}+\frac{v_{k}{\left(t\right)}^{2}}{2\mu g}$$

where $t_{k}^{r}$ denotes the reaction time of the driver where $t_{k}^{r}=1.45\;s$ for drivers in cars and $t_{k}^{r}=0.26\;s$ for drivers in trucks [81]. μ and g denote the friction coefficient and gravitational constant.

Once the driver makes a decision to change lane, the LCP begins with a direction towards the target lane with an angle of $\theta_{n}$, as demonstrated in Figure 3A. The formulation of $\theta_{n}$ is presented as follows:

(17) $$\theta_{n}=\max\left(\theta_{\min},\min\left(\theta_{\max},\theta_{c}\right)\right)\;$$

(18) $$\theta_{c}=\frac{180}{\pi }\arctan\frac{W}{{\hat{s}}_{n}^{2}\left(t\right)}$$

where $\theta_{\max}$ and $\theta_{\min}$ represent the upper and lower bounds of the lane-changing angle, respectively. W denotes the lane width. Let $y_{n}\left(t\right)$ denote the lateral location of vehicle n at time t, where the middle position of the inner lane is the origin. After $\theta_{n}$ is determined, the LCP is described in a recursive way, as shown in Table 1. During LCP, the inter-lane dependency is negligible such that λ=0. In Algorithm 1, the scenario is taken as an example where vehicle n is changing from the inner to outer lane.

**Algorithm 1** Recursive evaluation for LCP.Let lateral location of vehicle n in LCP $y_{n}\left(t\right)=0$, T denotes the time step. **while** $y_{n}\left(t\right)<W$ $\Delta s_{n}=v_{n}\left(t\right)T\sin\theta_{n}$, $x_{n}\left(t+T\right)=x_{n}\left(t\right)+v_{n}\left(t\right)T\cos\theta_{n}$, $y_{n}\left(t\right)=y_{n}\left(t\right)+\Delta s_{n}$; **if** $y_{n}\left(t\right)\leq \frac{W}{2}$  $v_{n}\left(t+T\right)=v_{n}\left(t\right)+a_{n}\left(t\right)T$, $a_{n}=a_{n}\left(v_{n}\left(t\right),x_{n}\left(t\right),{\hat{s}}_{n}^{1}\left(t\right),\Delta {\hat{v}}_{n}^{1}\left(t\right)\right)$, $s_{n}^{1}\left(t\right)=\Delta x_{n}^{1}\left(t\right)-l_{n-1}$, $\Delta v_{n}^{1}\left(t\right)=v_{n-1}\left(t\right)-v_{n}\left(t\right)\cos\theta_{n}$;  $v_{n+1}\left(t+T\right)=v_{n+1}\left(t\right)+a_{n+1}\left(t\right)T$, $a_{n+1}\left(t\right)=a_{n+1}\left(v_{n+1}\left(t\right),x_{n+1}\left(t\right),{\hat{s}}_{n+1}^{1}\left(t\right),\Delta {\hat{v}}_{n+1}^{1}\left(t\right)\right)$, $s_{n+1}^{1}\left(t\right)=\Delta x_{n+1}^{1}\left(t\right)-l_{n}\cos\theta_{n}$,  $\Delta v_{n+1}^{1}\left(t\right)=v_{n}\left(t\right)\cos\theta_{n}-v_{n+1}\left(t\right)$;  $v_{k}\left(t+T\right)=v_{k}\left(t\right)+a_{k}\left(t\right)T$, $a_{k}\left(t\right)=a_{k}\left(v_{k}\left(t\right),x_{k}\left(t\right),{\hat{s}}_{k}^{1}\left(t\right),\Delta {\hat{v}}_{k}^{1}\left(t\right)\right)$, $s_{k}^{1}\left(t\right)=\Delta x_{k}^{1}\left(t\right)-l_{k-1}$, $\Delta v_{k}^{1}\left(t\right)=v_{k-1}\left(t\right)-v_{k}\left(t\right)$; **else**
  $v_{n}\left(t+T\right)=v_{n}\left(t\right)+a_{n}\left(t\right)T$, $a_{n}\left(t\right)=a_{n}\left(v_{n}\left(t\right),x_{n}\left(t\right),{\hat{s}}_{n}^{2}\left(t\right),\Delta {\hat{v}}_{n}^{2}\left(t\right)\right)$, $s_{n}^{2}\left(t\right)=\Delta x_{n}^{2}\left(t\right)-l_{k-1}$, $\Delta v_{n}^{2}\left(t\right)=v_{k-1}\left(t\right)-v_{n}\left(t\right)\cos\theta_{n}$;  $v_{n+1}\left(t+T\right)=v_{n+1}\left(t\right)+a_{n+1}\left(t\right)T$, $a_{n+1}\left(t\right)=a_{n+1}\left(v_{n+1}\left(t\right),x_{n+1}\left(t\right),{\hat{s}}_{n+1}^{1}\left(t\right),\Delta {\hat{v}}_{n+1}^{1}\left(t\right)\right)$, $s_{n+1}^{1}\left(t\right)=\Delta x_{n+1}^{1}\left(t\right)-l_{n-1}$,  $\Delta v_{n+1}^{1}\left(t\right)=v_{n-1}\left(t\right)-v_{n+1}\left(t\right)$;  $v_{k}\left(t+T\right)=v_{k}\left(t\right)+a_{k}\left(t\right)T$, $a_{k}\left(t\right)=a_{k}\left(v_{k}\left(t\right),x_{k}\left(t\right),{\hat{s}}_{k}^{1}\left(t\right),\Delta {\hat{v}}_{k}^{1}\left(t\right)\right)$, $s_{k}^{1}\left(t\right)=\Delta x_{k}^{1}\left(t\right)-l_{n}\cos\theta_{n}$, $\Delta v_{k}^{1}\left(t\right)=v_{n}\left(t\right)\cos\theta_{n}-v_{k}\left(t\right)$ **end**  t=t+T; **end**

Once the lane-changing of the subject vehicle is finished, it should be noted that vehicle-following patterns of the five vehicles (subject vehicle and four surrounding vehicles, see Figure 3) vary after LCP.

### 3.3. SSM-Based Crash Risk

The studies on SSM can date back to early 1970s [82] and considerable achievements have been made in this field since then. SSMs are typically estimated using the preceding and following vehicles’ speeds and/or accelerations, and their spacing, which are derived from vehicle trajectory data based on recorded videos and/or microscopic traffic simulation results. SSMs fall into three categories: time-based SSMs, deceleration-based SSMs, and energy-based SSMs. The first two appear to be the most widely used. Time-based SSMs measure the time proximity to a crash occurrence, such as TTC [83]. Instead, deceleration-based SSMs focus on how vehicles’ deceleration can prevent crash occurrence.

The most common deceleration-based SSM is DRAC, which was initially proposed by Cooper and Ferguson to determine the severity of a traffic conflict [84]. DRAC is the minimum deceleration rate required for a vehicle to avoid collision with a preceding vehicle. The formulation of DRAC is based on the assumption that the following vehicle takes evasive actions while the preceding vehicle retains its speed and direction. It was also extended to another form which is incorporated with driver’s reaction times of the different types of following vehicles (i.e., car and truck) [81]. Compared to the time-based SSMs, the DRAC could reflect the evasive action of the following vehicle to stop in a timely manner or reach the corresponding speed of the leading vehicle and thereby avoid a crash [85]. Besides, the effectiveness of DRAC has been validated via comparison with the observed crash frequency [51,85].

The crash risk between two vehicles at the time $R_{n}\left(t\right)$ is defined as the probability that the instantaneous DRAC between two vehicles exceeds the maximum available deceleration rate (MADR), as Equation (19) indicates:

(19) $$R_{n}\left(t\right)=\mathrm{Pr}\left(DRAC_{n}\left(t\right)>MADR_{n}\right),\;$$

where $MADR_{n}$ denotes MADR of vehicle n. MADR is associated with various factors, such as pavement condition and vehicle type. In this study, the MADR at the dry pavement condition is considered and is assumed to only depend on the vehicle type. MADR follows a truncated normal distribution and hence we have

(20) $$R_{n}\left(t\right)=\left\{\begin{array}{ll} 0 & DRAC_{n}\left(t\right)\leq M_{n}^{low}\\ \frac{\Phi \left(M_{n}^{\mu },{\left(M_{n}^{\sigma }\right)}^{2};DRAC_{n}\left(t\right)\right)-\Phi \left(M_{n}^{\mu },{\left(M_{n}^{\sigma }\right)}^{2};M_{n}^{low}\right)}{\Phi \left(M_{n}^{\mu },{\left(M_{n}^{\sigma }\right)}^{2};M_{n}^{up}\right)-\Phi \left(M_{n}^{\mu },{\left(M_{n}^{\sigma }\right)}^{2};M_{n}^{low}\right)} & M_{n}^{low}<DRAC_{n}\left(t\right)<M_{n}^{up}\\ 1 & DRAC_{n}\left(t\right)\geq M_{n}^{up} \end{array}\right.$$

where $\Phi \left(M_{n}^{\mu },{\left(M_{n}^{\sigma }\right)}^{2};\cdot \right)$ denotes the distribution density function for the normal distribution where $M_{n}^{\mu }$ and $M_{n}^{\sigma }$ are the mean and standard deviation for $MADR_{n}$, respectively. $M_{n}^{up}$ and $M_{n}^{low}$ denote the upper and lower limits for the truncated normal distribution of $MADR_{n}$, respectively. $M_{n}^{\mu }$, $M_{n}^{\sigma }$, $M_{n}^{up}$, and $M_{n}^{low}$ for car and truck [81,86] are listed in Table 1.

DRAC is defined as the minimum deceleration rate of the following vehicle to stop in a timely manner behind the preceding vehicle as follows:

(21) $$DRAC_{n}\left(t\right)=\frac{0.5\min{\left\{0,\Delta v_{n}^{1}\left(t\right)\right\}}^{2}}{s_{n}^{1}\left(t\right)+\Delta v_{n}^{1}\left(t\right)t_{n}^{r}}$$

Equation (27) gives the DRAC of vehicle n at time t in the CF scenario where the driver’s reaction time $t_{n}^{r}$ is considered.

In the LC scenario, five vehicles (subject vehicle n and four surrounding vehicles in Figure 3B) are involved. Since DRAC describes the risk of following a vehicle colliding with the preceding vehicle, DRACs of vehicle n and its following vehicles on two lanes, vehicle n+1 and vehicle k, are evaluated. Figure 3B describes the scenario where vehicle n is changing from the inner to the outer lane and we have

(22) $$DRAC_{n+1}\left(t\right)=\left\{\begin{array}{l} DRAC_{n+1}^{\left(1\right)}\left(t\right)\;\;\;\;\;y_{n}\in \left[-0.5W+\frac{w_{n}}{2},y_{n+1}\left(t\right)+\frac{w_{n+1}}{2}+l_{n}\sin\theta_{n}+\frac{w_{n}}{2}\cos\theta_{n}\right]\\ DRAC_{n+1}^{\left(2\right)}\left(t\right)\;\;\;\;\;y_{n}\in \left(y_{n+1}\left(t\right)+\frac{w_{n+1}}{2}+l_{n}\sin\theta_{n}+\frac{w_{n}}{2}\cos\theta_{n}\left.,1.5W-\frac{w_{n}}{2}\right]\right. \end{array}\right.$$

where

(23) $$DRAC_{n+1}^{\left(1\right)}\left(t\right)=\frac{0.5\min{\left\{0,v_{n}\left(t\right)\cos\theta_{n}-v_{n+1}\left(t\right)\right\}}^{2}}{x_{n}\left(t\right)-x_{n+1}\left(t\right)-l_{n}\cos\theta_{n}-\frac{w_{n}}{2}\sin\theta_{n}+\left(v_{n}\left(t\right)\cos\theta_{n}-v_{n+1}\left(t\right)\right)t_{n+1}^{r}}$$

(24) $$DRAC_{n+1}^{\left(2\right)}\left(t\right)=\frac{0.5\min{\left\{0,v_{n-1}\left(t\right)-v_{n+1}\left(t\right)\right\}}^{2}}{x_{n-1}\left(t\right)-x_{n+1}\left(t\right)-l_{n-1}+\left(v_{n-1}\left(t\right)-v_{n+1}\left(t\right)\right)t_{n+1}^{r}}$$

$DRAC_{n+1}^{\left(1\right)}$ indicates the risk of vehicle n+1 colliding with vehicle n if the rear-end of vehicle n is still on the inner lane. $DRAC_{n+1}^{\left(2\right)}$ indicates the risk of vehicle n+1 colliding with vehicle n−1 if the rear-end of vehicle n leaves the inner lane and vehicle n−1 becomes the preceding vehicle of vehicle n+1. For vehicle k, we have

(25) $$DRAC_{k}\left(t\right)=\left\{\begin{array}{l} DRAC_{k}^{\left(1\right)}\left(t\right)\;\;\;\;\;y_{n}\in \left[\left.-0.5W+\frac{w_{n}}{2},y_{k}\left(t\right)-\frac{w_{k}}{2}-\frac{w_{n}}{2}\cos\theta_{n}\right)\right.\\ DRAC_{k}^{\left(2\right)}\left(t\right)\;\;\;\;\;y_{n}\in \left[y_{k}\left(t\right)-\frac{w_{k}}{2}-\frac{w_{n}}{2}\cos\theta_{n},1.5W-\frac{w_{n}}{2}\right] \end{array}\right.$$

where

(26) $$DRAC_{k}^{\left(1\right)}\left(t\right)=\frac{0.5\min{\left\{0,v_{k-1}\left(t\right)-v_{k}\left(t\right)\right\}}^{2}}{x_{k-1}\left(t\right)-x_{k}\left(t\right)-l_{k-1}+\left(v_{k-1}\left(t\right)-v_{k}\left(t\right)\right)t_{k}^{r}}$$

(27) $$DRAC_{k}^{\left(2\right)}\left(t\right)=\frac{0.5\min{\left\{0,v_{n}\left(t\right)\cos\theta_{n}-v_{k}\left(t\right)\right\}}^{2}}{x_{n}\left(t\right)-x_{k}\left(t\right)-l_{n}\cos\theta_{n}-\frac{w_{n}}{2}\sin\theta_{n}+\left(v_{n}\left(t\right)\cos\theta_{n}-v_{k}\left(t\right)\right)t_{k}^{r}}$$

$DRAC_{k}^{\left(1\right)}$ indicates the risk of vehicle k colliding with vehicle k−1 if the head of vehicle n has not reached the outer lane. $DRAC_{n+1}^{\left(2\right)}$ indicates the risk of vehicle k colliding with vehicle n if the head of vehicle n occupies the outer lane and vehicle n becomes the preceding vehicle of k. 

For vehicle n, if $\frac{1}{2}\left(w_{n-1}+w_{k-1}\right)+w_{n}\cos\theta_{n}\leq W$, we have

(28) $$DRAC_{n}\left(t\right)=\left\{\begin{array}{ll} DRAC_{n}^{\left(1\right)}\left(t\right) & y_{n}\in \left[\left.-0.5W+\frac{w_{n}}{2},y_{n-1}\left(t\right)+\frac{w_{n-1}}{2}+\frac{w_{n}}{2}\cos\theta_{n}\right)\right.\\ 0 & y_{n}\in \left[y_{n-1}\left(t\right)+\left.\frac{w_{n-1}}{2}+\frac{w_{n}}{2}\cos\theta_{n},y_{k-1}\left(t\right)-\frac{w_{k-1}}{2}-\frac{w_{n}}{2}\cos\theta_{n}\right)\right.\\ DRAC_{n}^{\left(2\right)}\left(t\right) & y_{n}\in \left[y_{k-1}\left(t\right)-\frac{w_{k-1}}{2}-\frac{w_{n}}{2}\cos\theta_{n},1.5W-\frac{w_{n}}{2}\right] \end{array}\right.$$

Otherwise

(29) $$DRAC_{n}\left(t\right)=\left\{\begin{array}{ll} DRAC_{n}^{\left(1\right)}\left(t\right) & y_{n}\in \left[\left.-0.5W+\frac{w_{n}}{2},y_{n-1}\left(t\right)+\frac{w_{n-1}}{2}+\frac{w_{n}}{2}\cos\theta_{n}\right)\right.\\ \max\left\{DRAC_{n}^{\left(1\right)}\left(t\right),DRAC_{n}^{\left(2\right)}\left(t\right)\right\} & y_{n}\in \left[y_{n-1}\left(t\right)+\left.\frac{w_{n-1}}{2}+\frac{w_{n}}{2}\cos\theta_{n},y_{k-1}\left(t\right)-\frac{w_{k-1}}{2}-\frac{w_{n}}{2}\cos\theta_{n}\right)\right.\\ DRAC_{n}^{\left(2\right)}\left(t\right) & y_{n}\in \left[y_{k-1}\left(t\right)-\frac{w_{k-1}}{2}-\frac{w_{n}}{2}\cos\theta_{n},1.5W-\frac{w_{n}}{2}\right] \end{array}\right.$$

where

(30) $$DRAC_{n}^{\left(1\right)}\left(t\right)=\frac{0.5\min{\left\{0,v_{n-1}\left(t\right)-v_{n}\left(t\right)\cos\theta_{n}\right\}}^{2}}{x_{n-1}\left(t\right)-x_{n}\left(t\right)-l_{n-1}-\frac{w_{n}}{2}\sin\theta_{n}+\left(v_{n-1}\left(t\right)-v_{n}\left(t\right)\cos\theta_{n}\right)t_{n}^{r}}$$

(31) $$DRAC_{n}^{\left(2\right)}\left(t\right)=\frac{0.5\min{\left\{0,v_{k-1}\left(t\right)-v_{n}\left(t\right)\cos\theta_{n}\right\}}^{2}}{x_{k-1}\left(t\right)-x_{n}\left(t\right)-l_{k-1}-\frac{w_{n}}{2}\sin\theta_{n}+\left(v_{k-1}\left(t\right)-v_{n}\left(t\right)\cos\theta_{n}\right)t_{n}^{r}}$$

$DRAC_{n}^{\left(1\right)}$ indicates the risk of vehicle n colliding with vehicle n−1 while $DRAC_{n}^{\left(2\right)}$ indicates the risk of vehicle n colliding with vehicle k. As vehicle n moves from inner to outer lane, the crash target of vehicle n transfers from vehicle n−1 to vehicle k. If the lateral spacing between vehicle n−1 and vehicle k is wide, there will be no crash risk for vehicle n at a certain time, as Equation (28) presents. If the lateral spacing between vehicle n−1 and vehicle k is narrow, there will be a risk for vehicle n to collide with both vehicle n−1 and vehicle k at a certain time, as Equation (29) presents.

The core of the derivation of DRAC is similar while vehicle n is changing from the outer to inner lane and is not explicated in this paper.

## 4. Scenarios and Simulations

**Figure 4:**
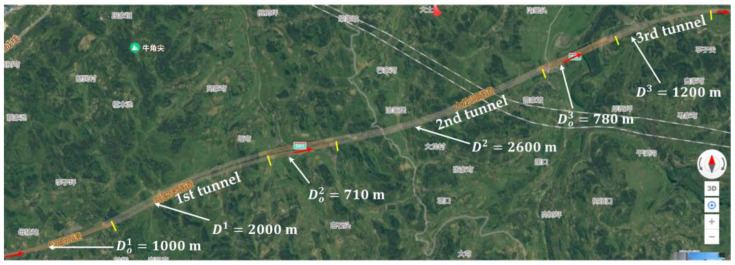
Studied serial tunnels for numerical examples.

In this section, real-world-based numerical examples are presented from a section of serial tunnels on the two-lane freeway G65, Chongqing, China, as demonstrated in Figure 4. The serial tunnels consist of three tunnels, where the tunnel lengths are $D^{1}=2000\;m$, $D^{2}=2600\;m$, and $D^{3}=1200\;m$, respectively. The distances between two tunnels are $D_{o}^{2}=710\;m$ and $D_{o}^{3}=780\;m$, respectively. The road is designed with solid lane marking stretching from 150 m ahead of the entrance portal of the 1st tunnel to 100 m behind of the exit portal of the 3rd tunnel, where LCs are prohibited for drivers to make. In other parts of serial tunnels, LCs are discretionary with dash lane marking. The widths for both lanes are 3.75 m. The noon or afternoon is the time for the basic scenario where exterior environmental luminance $L_{0}=6000\;\mathrm{cd}/m^{2}$.

In the tunnel lighting design standard of China [77], a stepwise curve method is used for zone luminance. For the ith tunnel the entrance zone is further divided into two portions of the threshold zone ${\mathbb{Z}}_{th1}^{i}$ and ${\mathbb{Z}}_{th2}^{i}$, and three portions of the transition zone ${\mathbb{Z}}_{tr1}^{i}$, ${\mathbb{Z}}_{tr2}^{i}$, and ${\mathbb{Z}}_{tr3}^{i}$. The exit zone is further divided into two portions ${\mathbb{Z}}_{ex1}^{i}$ and ${\mathbb{Z}}_{ex2}^{i}$. The ending positions of these portions are $x_{th1}^{i}$, $x_{th2}^{i}$, $x_{tr1}^{i}$, $x_{tr2}^{i}$, $x_{tr3}^{i}$, $x_{ex1}^{i}$, and $x_{ex2}^{i}$, respectively, where $x_{tr3}^{i}=x_{et}^{i}$ and $x_{ex2}^{i}=x_{et}^{i}$. The lengths of these portions are $D_{th1}^{i}$, $D_{th2}^{i}$, $D_{tr1}^{i}$, $D_{tr2}^{i}$, $D_{tr3}^{i}$, $D_{ex1}^{i}$, and $D_{ex2}^{i}$, respectively. The location at 1000 m ahead of the entrance portal of the 1st tunnel is denoted as x=0. Hence, $x_{o}^{1}=D_{o}^{1}=1000\;m$. The lengths are $D_{th1}^{i}=D_{th2}^{i}=42\;m$, $D_{tr1}^{i}=72\;m$, $D_{tr2}^{i}=89\;m$, $D_{tr3}^{i}=133\;m$, $D_{ex1}^{i}=30\;m$, and $D_{ex2}^{i}=30\;m$ for all three tunnels. $D_{in}^{1}=1562\;m$, $D_{in}^{2}=2162\;m$, $D_{in}^{3}=762\;m$.

The first portion of the threshold zone has a constant luminance level $L_{th1}^{i}$ which is the base for luminance design in the entrance zone. The luminance in the second portion of the threshold zone is decreasing with the average value of $L_{th2}^{i}=0.5L_{th1}^{i}$. The luminance in three portions of the transition zone is gradually decreasing with average values of $L_{tr1}^{i}=0.15L_{th1}^{i}$, $L_{tr2}^{i}=0.05L_{th1}^{i}$, and $L_{tr3}^{i}=0.02L_{th1}^{i}$, respectively. If $L_{tr3}^{i}\leq L_{in}$, $L_{tr3}^{i}=L_{in}^{i}$ in the design. The luminance in two portions of the exit zone is gradually increasing based on $L_{in}^{i}$ with average values of $L_{ex1}^{i}=3L_{in}^{i}$ and $L_{ex2}^{i}=5L_{in}^{i}$, respectively.

### 4.1. Scenarios

**Figure 5:**
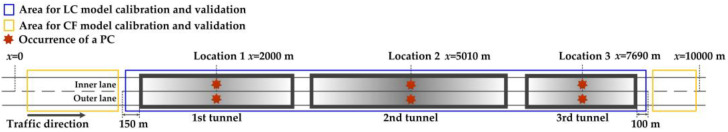
Locations of PC occurrences and areas for model validation.

There are six possible locations of PC occurrences investigated, consisting of middle positions in all three tunnels on both inner and outer lanes, as shown in Figure 5.

An amount of 3000 cd/m^2^ of the exterior environmental luminance $L_{0}$ is always the fundamental parameter of tunnel lighting design in China for all portions [77]. The two basic luminance levels are $L_{th1}^{i}=0.025\times 3000=75\;\mathrm{cd}/m^{2}$ and $L_{in}^{i}=1.0\;\mathrm{cd}/m^{2}$. The “black hole” and “white hole” effects are very likely to happen for drivers in the basic scenario where $L_{0}=6000\;\mathrm{cd}/m^{2}$, as presented in Scenario 0.

**Scenario** **0.** *The luminance levels of all portions are* $L_{th1}=75\;cd/m^{2}$ *,* $L_{th2}=37.5\;cd/m^{2}$ *,* $L_{tr1}=11.25\;cd/m^{2}$ *,* $L_{tr2}=3.75\;cd/m^{2}$ *,* $L_{tr3}=1.5\;cd/m^{2}$ *,* $L_{in}=1.0\;cd/m^{2}$ *,* $L_{ex1}=3.0\;cd/m^{2}$ *,* $L_{ex2}=5.0\;cd/m^{2}$ *, respectively.*

#### 4.1.1. Simulation Time Step Comparison

We determined the appropriate time step in the simulation for the sake of efficiency based on Scenario 0. The alternative time steps are 0.1 and 1.0 s. To make a fast decision on the time step, we reduce the scale of the car platoon and the duration of PC. In the simulation, 200 vehicles with 20% trucks run from the beginning of the section. The average values of initial spacings and desired speeds are 100 m and 80 km/h, respectively. The PC occurs on the outer lane of the middle positions in the 2nd tunnel (i.e., Location 2 in Figure 5). The duration for PC is set as 10 min.

#### 4.1.2. Model Validation

The LMTID-based simulation is validated via the comparison with the experimental observation of real traffic in studied serial tunnels. In the simulation, the location and duration of PC occurrence are set in terms of the video records. In addition, the video records when no PC occurs are used for validation as well.

The CF model is validated in the area where the road is designed with solid lane marking and LCs are prohibited for drivers to make, as presented in Figure 5. Since the sequence of cars and the result of the CF model are deterministic, a platoon-based comparison between real traffic and a simulation is feasible, such as the distribution of travel times and RMSE of the trajectory’s speed profile [87] in car platoon. The real trajectories of cars are constructed via the array of roadside cameras with 150 m spacing on the tunnel wall (third type of camera in Figure 2). Car speeds and times crossing the camera view (regarded as a virtual cross-sectional detector) are automatically captured by each camera. The cars’ number plates and types (i.e., car or truck) are manually recorded in the video replay of each camera due to the narrow side of the camera and low illuminance in the interior zone of the tunnel. Based on the sequences of matched cars’ number plates, types, times, and speeds crossing the virtual cross-sectional detectors in all cameras, trajectories of car platoons can be formed. In the simulation for the CF model, the luminance levels along serial tunnels should have been measured in field. However, we did not grant the permission to measure the road surface luminance in the studied serial tunnels. Therefore, we use the designed tunnel lighting scheme (i.e., Scenario 0) to substitute for the real-world road surface luminance. In addition, the initial state of the simulation coincides with that in the beginning of the video records, including the locations, speeds, and types of all vehicles.

The LC model is validated in the area at two ends of the section of serial tunnels where LCs are discretionary with dash lane marking and covered by a panoramic camera (second type of camera in Figure 2), as presented in Figure 5. Due to the stochasticity of LCs, platoon-based validation is insufficient. Instead, we use macroscopic traffic variables of real traffic and a simulation for comparison, such as a density-flow diagram and LC frequency [75]. The experimental observations are recorded by panoramic camera. The flow and density are derived from the vehicle number of each lane for every second. In the simulation for the LC model, the initial state of the simulation coincides with that in the beginning of the video records, including the locations, speeds, and types of all vehicles.

Besides, the lighting-related behavioral parameters ($T_{a}$, $b_{a}$, $k_{1}$, $k_{2}$, and $b_{in}$) are calibrated via naturalistic driving experiments for drivers in other different tunnels, which are explicated in Appendix A.

#### 4.1.3. SC Risk under Current Situation and Countermeasures

Firstly, we investigate the features of SC risks over time for different locations of PC occurrence in serial tunnels. Therefore, PC occurs in parallel at all six locations of Figure 5 in Scenario 0.

Then, the effectiveness of countermeasures is investigated. For the scenario under ATLC, the luminance levels in all portions adapt to the exterior environmental luminance 6000 cd/m^2^, $L_{th1}^{i}=0.025\times 6000=150\;\mathrm{cd}/m^{2}$, $L_{in}^{i}=2.5\;\mathrm{cd}/m^{2}$ [77], as presented in Scenario 1. For the scenario under ASLG, CVs will start to decelerate 1 km ahead of the tail of the queue if they decide to select the lane where PC occurs. The desired speed $\tilde{V}\left(\cdot \right)$ is assumed to be reduced by 40%. Otherwise, CVs will change to the adjacent lane 1 km ahead of the tail of the queue or 1 km ahead of the beginning of the solid lane marking. The proportions of CVs are 30%, 60%, and 90% for Scenarios 2, 3, and 4, respectively. Finally, the countermeasure combining ATLC and ASLG is proposed with 30% CVs, as Scenario 5 presents. In these scenarios, the locations of PC occurrences are the same, the inner lane of middle positions in 2nd tunnel (i.e., Location 2 in Figure 5).

**Scenario** **1.** *(ATLC) The luminance levels of all portions are* $L_{th1}=150\;cd/m^{2}$ *,* $L_{th2}=75\;cd/m^{2}$ *,* $L_{tr1}=22.5\;cd/m^{2}$ *,* $L_{tr2}=7.5\;cd/m^{2}$ *,* $L_{tr3}=3.0\;cd/m^{2}$ *,* $L_{in}=2.5\;cd/m^{2}$ *,* $L_{ex1}=7.5\;cd/m^{2}$ *,* $L_{ex2}=12.5\;cd/m^{2}$ *, respectively. Other variables are the same as Scenario 0.*

**Scenario** **2.** *(30%ASLG) The proportion of CVs is 30%. The luminance levels of all portions are the same as Scenario 0*.

**Scenario** **3.** *(60%ASLG) The proportion of CVs is 60%. The luminance levels of all portions are the same as Scenario 0*.

**Scenario** **4.** *(90%ASLG) The proportion of CVs is 90%. The luminance levels of all portions are the same as Scenario 0*.

**Scenario** **5.** *(ATLC&30%ASLG) The proportion of CVs is 30%. The luminance levels of all portions are the same as Scenario 1*.

In the simulations, 2500 vehicles with 20% trucks run from the beginning of the section. The average values of initial spacings and desired speeds are 100 m and 80 km/h, respectively. The duration for PC is set as 60 min.

### 4.2. Simulations

In the simulation, the vehicle lengths for cars and trucks are 6 m and 12 m, respectively. The vehicle widths for cars and trucks are 1.8 m and 2.5 m, respectively. For the CF submodel and LCP in LMTID, The response coefficient of the relative speed between vehicles on the subject and adjacent lanes λ=0.6. The acceleration exponent β needs to be calibrated. For LCD in LMTID, the reaction times of the drivers in cars and trucks are 1.45 s and 0.26 s, respectively. The upper and lower bounds of the LC angle are $\theta_{\min}=5{}^{\circ}$ and $\theta_{\max}=20{}^{\circ}$, respectively. The friction coefficient μ=0.8 and the gravitational constant $g=9.8\;m/s^{2}$. The LC probabilities $\left[p_{1},p_{2},p_{3}\right]$ are the parameters that need to be calibrated. The minimum necessary spacing for LC $s_{\min}=5\;m$. For SC risk evaluation, the distribution parameters of MADR for cars and trucks are listed in Algorithm 1. The simulation is coded in MATLAB and run on a workstation.

For model validation, the simulation program is initialized according to the beginning frame of the video records, including the locations, speeds, and types of all vehicles. For the time step determination and SC risk evaluation, the simulation program is initialized by distributing vehicles randomly on the two lanes via the distribution of the spacings between two consecutive vehicles. The spacings and speeds yield to normal distribution. Whether a vehicle is a car or a truck, connected or unconnected vehicles are generated via Bernoulli distributions in terms of proportions, where no truck is generated on the inner lane initially.

In the program, we set an attribute set for each vehicle which includes vehicle No., lane No., serial No., vehicle angle, position, velocity, acceleration, and spacing. The vehicle’s serial No. remains unchangeable while other attributes will be updated during the course of simulation. In each time step, a CF calculation (i.e., Equations (11) and (12)) is accomplished to determine the vehicle movement on the subject lane. Then a judgment for LCD is made, whether a vehicle changes lanes or not (i.e., Equations (13) and (15)). If the LC rule is satisfied, the vehicle will start LCP and LCP is operated according to Algorithm 1. At the end of LCP, the vehicle has moved to the target lane and then it will move on adjacent lane in the next time step. After each time step, all vehicles’ attributes, i.e., lane, angle, position, velocity, acceleration, and spacing, are updated.

It should be noted that the initial state of the simulation can be set according to the real-time traffic flow data from the AVI camera (first type of camera in Figure 2) if the program is plugged into the traffic surveillance system in the freeway management center.

## 5. Results and Discussion

### 5.1. Simulation Time Step Determination

**Figure 6:**
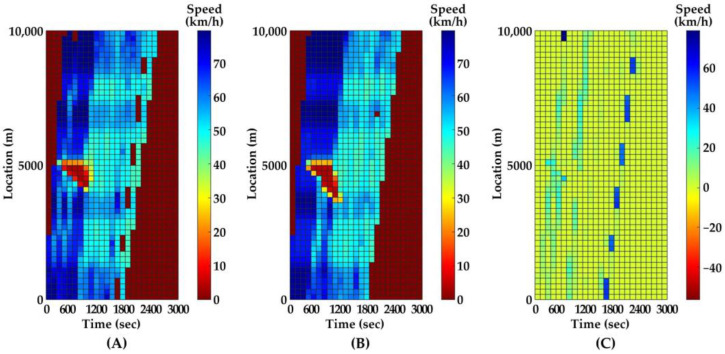
Mean speeds contour on inner lane. (**A**) time step is 0.1 s; (**B**) time step is 1.0 s; (**C**) difference between two time steps.

**Figure 7:**
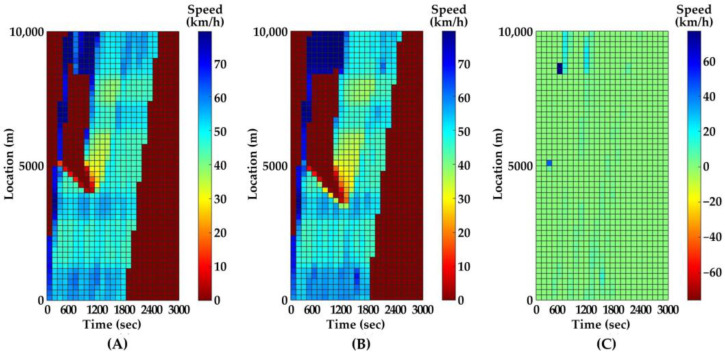
Mean speeds contour on outer lane. (**A**) time step is 0.1 s; (**B**) time step is 1.0 s; (**C**) difference between two time steps.

Since the vehicle trajectories are stochastic from the simulation, we cannot directly compare the trajectories between two time steps. Instead, we use the mean speed in a spatiotemporal interval as the comparison metric. The temporal and spatial intervals are 2 min and 200 m, respectively. After 100 parallel simulations, the medians of metrics in the two time steps (i.e., 0.1 and 1.0 s) and their difference are presented as contours in Figure 6 and Figure 7.

As Figure 6 and Figure 7 indicate, the predicted traffic flow evolutions after PC occurs with different time steps are similar. However, the average running times in 100 parallel simulations with 0.1- and 1.0-min time steps are 5.4 and 0.6 min, respectively. As a result of a lower computational capacity requirement and a higher efficiency, 1.0 s is determined as the time step for the simulation in all following scenarios. In addition, the relatively faster speed of the program with a 1.0 s time step makes our approach more feasible to plug into the traffic surveillance system in the freeway management center.

### 5.2. Model Validation

For the CF part of LMTID, there are many parameters that need to be calibrated. Due to limited observation data in our study, the most sensitive parameter is selected to calibrate, and the values of others come from the results of previous studies observed in China. The acceleration exponent β is often presented as the most sensitive parameter to affect the results [59,60,61] and will be validated in this paper. The alternative values for β are 2 and 4. The values of ${\tilde{h}}_{n}$, $s_{n}^{\mathrm{jam}}$, $v_{n}^{0}$, $a_{n}^{\max}$, and $a_{n}^{\mathrm{comf}}$ for four types of vehicle-following patterns are listed in Table 2 [23].

**Figure 8:**
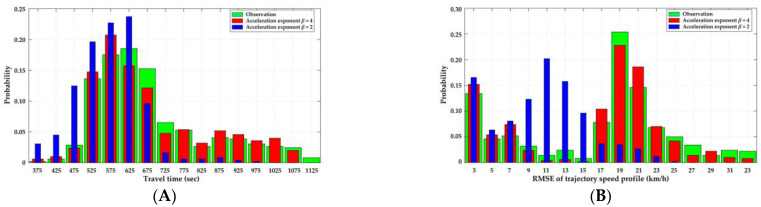
Validation for CF model comparing the observation and simulation. (**A**) comparison of travel time distribution in platoon; (**B**) comparison of RMSE of trajectory’s speed profile distribution in platoon.

A 30 min video record after a PC occurs is available for us to validate the CF model. The PC was a slight rear-end crash between two cars, and hence the duration of PC lasted for about 18.4 min. The PC is located on the outer lane and 784 m to the entrance of the first tunnel. We compare the distributions of travel times and the RMSE of the trajectory’s speed profile in car platoons on both lanes. The latter measure describes the magnitude of an individual vehicle’s speed oscillation. The calculation of RMSE of the trajectory’s speed profile can be found in [87]. The comparisons among observations and simulations with different values of acceleration exponent β are presented in Figure 8.

The results show that β=4 is appropriate for the CF model and the CF model roughly corresponds with the experimental observation result. The error may result from the narrowness effect of the tunnel wall and visual fatigue due to low illuminance [88] when drivers travel through serial tunnels. These factors affecting driving behavior need to be considered in LMTID in future research. In addition, distributions of travel times and the RMSE of the trajectory’s speed profile in car platoons are bimodal where the second peak presents the vehicles congested upstream of the PC.

**Figure 9:**
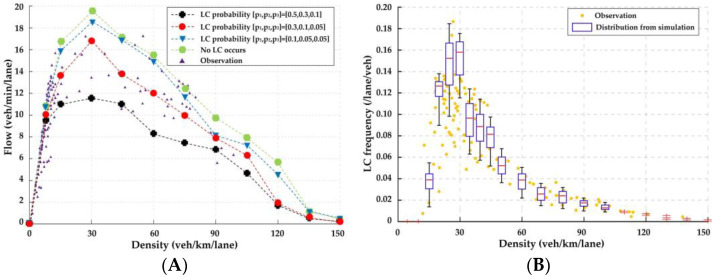
Validation for LC model comparing the observation and simulation. (**A**) flow-density comparison; (**B**) LC frequency comparison.

For the LC part of LMTID, LC probability is the most representative parameter that needs to be calibrated. The same video record with the CF model validation is used, with other video records when no crash occurs supplemented. We compare the flow-density diagram and LC frequency on both lanes. Since the results from the LC model are stochastic, we use the distribution of 100 parallel simulation results or the median of the distribution. The comparisons among observation and simulations with different values of LC probabilities $\left[p_{1},p_{2},p_{3}\right]$ are presented in Figure 9.

In the flow-density comparison, we compare the median of 100 parallel simulation results with the observation. $\left[p_{1},p_{2},p_{3}\right]=\left[0.3,0.1,0.05\right]$ is consistent with the experimental observation. The results also imply that some of the drivers seem somewhat aggressive when changing lanes, which coincides with the driving characteristics of Chinese drivers in the studies of driving cultures [73,74].

In the LC frequency comparison, we compare the distribution of 100 parallel simulation results with the observation. Figure 9A demonstrates the results of the simulation agree well with the observation. As traffic density increases, LC frequency first increases and then decreases. This is because that vehicles change lanes for better driving conditions with the increase of traffic density and they find it difficult to change lanes with a small spacing to the surrounding vehicles on both lanes at a high density condition. 

It should be noted that, to obtain more accurate results, more video records after PC occurs are needed to calibrate the LMTID. When the video record covering both PC and SC becomes available, DRAC-based SC risk in our study will be verified.

### 5.3. Features of SC Risks over Time for Different PC Locations in Serial Tunnels

**Figure 10:**
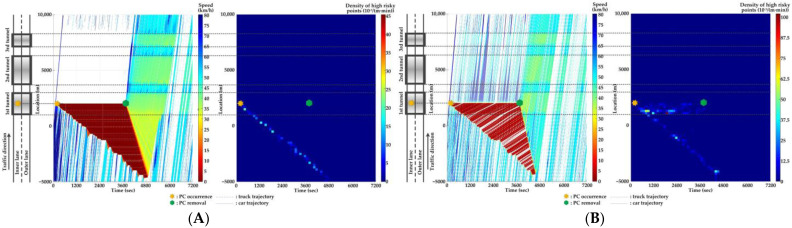
Lane-based evolutions for traffic flow (**left**) and crash risk (**right**) after PC occurs on the inner lane of Location 1. (**A**) inner lane; (**B**) outer lane.

**Figure 11:**
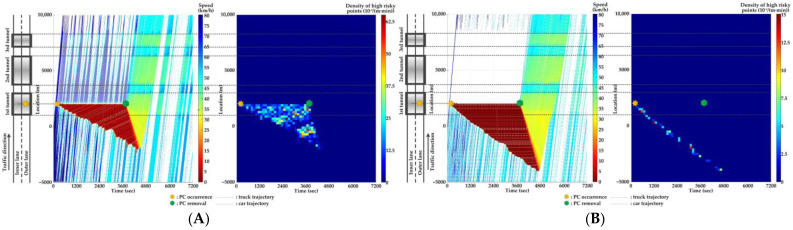
Lane-based evolutions for traffic flow (**left**) and crash risk (**right**) after PC occurs on the outer lane of Location 1. (**A**) inner lane; (**B**) outer lane.

**Figure 12:**
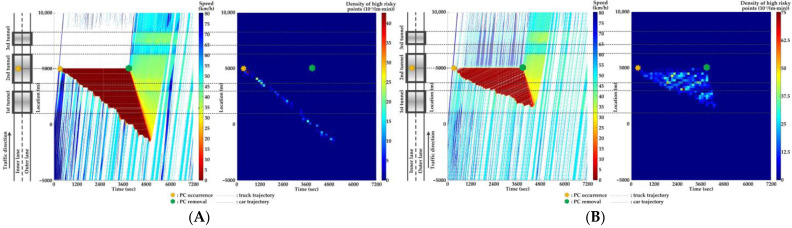
Lane-based evolutions for traffic flow (**left**) and crash risk (**right**) after PC occurs on the inner lane of Location 2. (**A**) inner lane; (**B**) outer lane.

**Figure 13:**
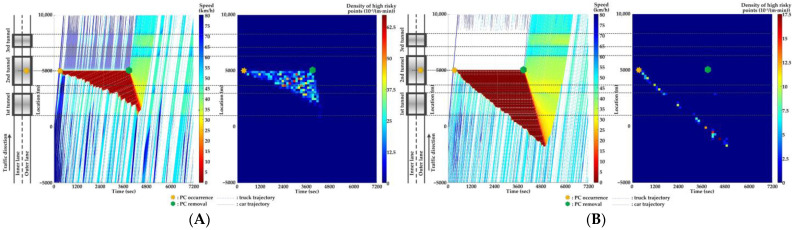
Lane-based evolutions for traffic flow (**left**) and crash risk (**right**) after PC occurs on the outer lane of Location 2. (**A**) inner lane; (**B**) outer lane.

**Figure 14:**
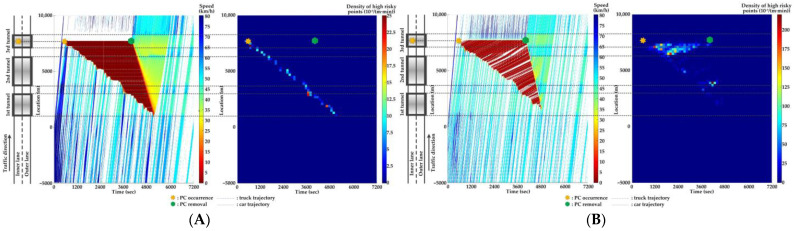
Lane-based evolutions for traffic flow (**left**) and crash risk (**right**) after PC occurs on the inner lane of Location 3. (**A**) inner lane; (**B**) outer lane.

**Figure 15:**
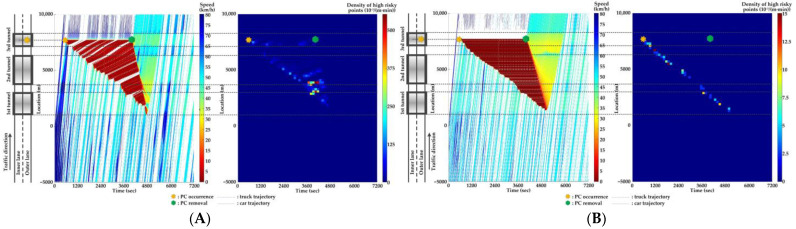
Lane-based evolutions for traffic flow (**left**) and crash risk (**right**) after PC occurs on the outer lane of Location 3. (**A**) inner lane; (**B**) outer lane.

For Scenario 0, we investigate the features of SC risks over time for different PC occurrence locations in serial tunnels. The high-risk points (HRPs) that are spatiotemporally distributed are aggregated for a better illustration of crash risk evolutions. A HRP is defined as $\left(t,x_{n}\left(t\right)\right)$ when $R_{n}\left(t\right)\geq 0.8$ for vehicle n. The temporal and spatial intervals for aggregation are 2 min and 200 m, respectively. Then, the density of HRPs can be obtained via the ratio of the aggregated number of HRPs to the area of the spatiotemporal interval. Traffic flow and crash risk evolutions for two lanes are presented in Figure 10, Figure 11, Figure 12, Figure 13, Figure 14 and Figure 15 when PC occurs on both lanes at six locations, respectively.

As Figure 10, Figure 11, Figure 12, Figure 13, Figure 14 and Figure 15 indicate, we can observe the followings.

- The stretching tail of the queue incurred by PC occurrence is always the high risky location over time, since the arriving vehicle has to brake to a halt before colliding with the tail of the queue.
- For the lane adjacent to the PC occurrence lane, it has even higher numbers of high-risk points due to the inter-lane dependency and low illumination inside the tunnel. The spatial range of HRPs stretches as the PC-incurred queue on the adjacent lane extends. As a result of the “black hole” and “white hole” effects, the density of HRPs is significantly high near tunnel portals in serial tunnels.
- If PC occurs at the rear location of serial tunnels, the density of SC risk on the PC occurrence lane is relatively low since drivers maintain a slower speed. However, the density of SC risk on the adjacent lane is higher with the stretching PC-incurred queue along serial tunnels, due to the inter-lane dependency and low illumination.
- Periodic oscillations of the traffic flow pattern are presented near tunnel portals where VAs are needed for drivers, demonstrated by the high density traffic flow departing from the PC occurrence location after PC removal.

### 5.4. Comparison of Countermeasures for SC Risk

**Figure 16:**
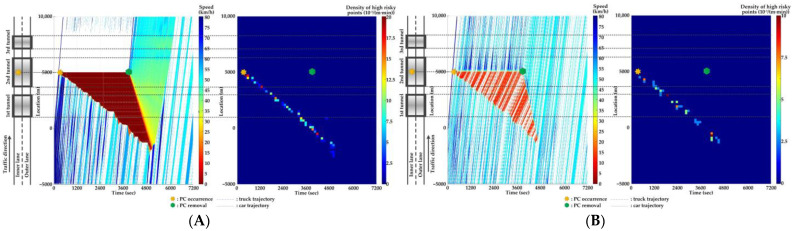
Lane-based evolutions for traffic flow (**left**) and crash risk (**right**) with ATLC after PC occurs on the inner lane of Location 2. (**A**) inner lane; (**B**) outer lane.

**Figure 17:**
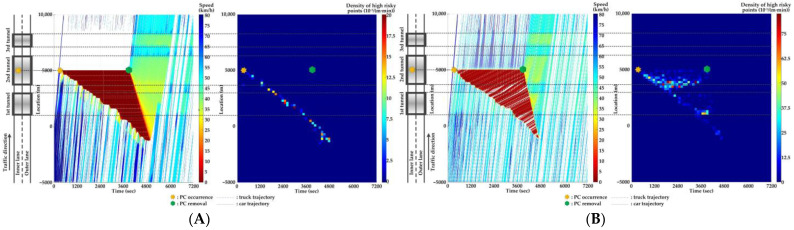
Lane-based evolutions for traffic flow (**left**) and crash risk (**right**) with 30%ASLG after PC occurs on the inner lane of Location 2. (**A**) inner lane; (**B**) outer lane.

**Figure 18:**
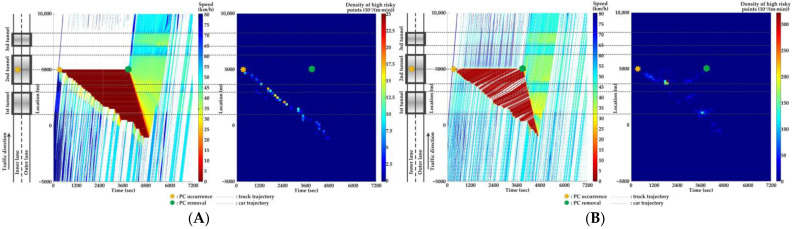
Lane-based evolutions for traffic flow (**left**) and crash risk (**right**) with 60%ASLG after PC occurs on the inner lane of Location 2. (**A**) inner lane; (**B**) outer lane.

**Figure 19:**
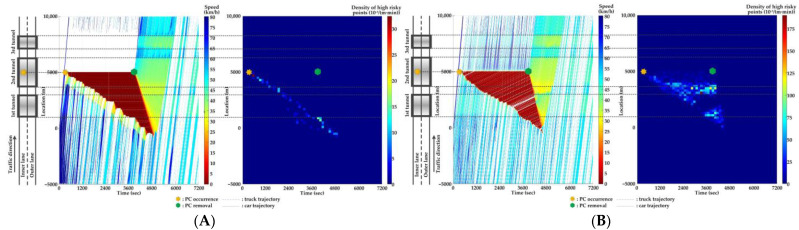
Lane-based evolutions for traffic flow (**left**) and crash risk (**right**) with 90%ASLG after PC occurs on the inner lane of Location 2. (**A**) inner lane; (**B**) outer lane.

**Figure 20:**
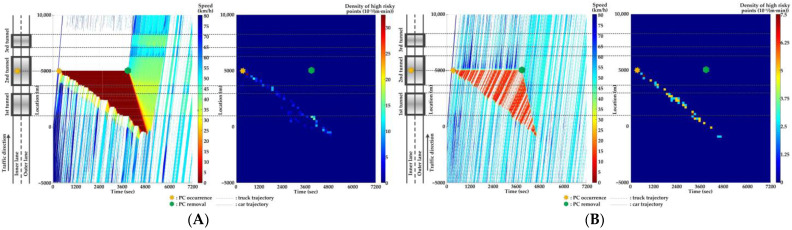
Lane-based evolutions for traffic flow (**left**) and crash risk (**right**) with ATLC&30%ASLG after PC occurs on the inner lane of Location 2. (**A**) inner lane; (**B**) outer lane.

Traffic flow and crash risk evolutions are compared with different countermeasures for SC risk from Scenarios 1 to 5 when PC occurs on the inner lane of Location 2, as presented in Figure 16, Figure 17, Figure 18, Figure 19 and Figure 20. Meanwhile, the effectiveness of countermeasures is compared with the scenario under no countermeasure, i.e., Figure 12.

As Figure 12, Figure 16, Figure 17, Figure 18, Figure 19 and Figure 20 indicate, we can observe the followings.

- In serial tunnels, creating good lighting conditions for drivers is more effective than advanced warnings for a part of drivers in CVs to mitigate the SC risk after PC occurrence.
- Since ATLC can successfully eliminate the “black hole” and “white hole” effects in terms of the daylight theoretically, the SC risk at the tail of the PC-incurred queue is alleviated when tail reaches the portal areas. However, the overall improvement on the PC occurrence lane is not observable. Meanwhile, ATLC can adjust the tunnel lighting inside the tunnel. It can considerably reduce the impact of inter-lane dependency and low illumination on crash risk along the adjacent lane of PC occurrence.
- ASLG for CVs manages to better alleviate the SC risk at the tail of the PC-incurred queue since CVs are able to immediately response to the hazardous traffic flow evolution from PC. However, a few new SC risky locations appear, resulting from the advanced deceleration of CVs whose drivers decide not to change to the adjacent lane. The increase of CV proportions raises the crash risk due to the advanced deceleration of CVs.
- ASLG fails to limit the crash risk along the adjacent lane of PC occurrence since it cannot eliminate the inter-lane dependency after it guides a part of CVs to the adjacent lane. As the proportion of CV rises, the locations and the density of crash risk along the adjacent lane significantly increase.
- A countermeasure combining ATLC with ASLG is promising since it can prevent the “black hole” and “white hole” effects near tunnel portals, inter-lane dependency, and low illumination inside the tunnel, and immediately inform drivers in CVs of the hazardous traffic flow evolution from PC.

## 6. Conclusions

To model and mitigate the secondary crash (SC) risk for serial tunnels on the freeway, this paper developed a traffic conflict approach where traffic turbulence is associated with primary crash (PC) occurrence and lighting condition variations along serial tunnels. In the approach, SC risk was quantified using surrogate safety measure (SSM) based on simulated vehicle trajectories after PC occurs via a lighting-related microscopic traffic model with inter-lane dependency (LMTID). CF and LC behaviors in LMTID were associated with location-heterogeneous lighting conditions along serial tunnels and traffic turbulence, which consists of shockwave after PC occurs, vehicle type heterogeneity, and inter-lane dependency. The deceleration rate to avoid the crash (DRAC) was utilized as a SSM to evaluate the SC risk. The numerical examples of a section of serial tunnels on a two-lane freeway were presented to validate the model, illustrate the SC risks over time for different PC locations and countermeasures, including adaptive tunnel lighting control (ATLC) for the present and advanced speed and lane-changing guidance (ASLG) for connected vehicles (CVs) in the future mobility.

The result demonstrates that the tail of the stretching queue on PC occurrence lane, the stretching section on adjacent lane of the PC-incurred queue, and areas near tunnel portals are the high-risk locations, due to the lack of immediate response to the hazardous traffic flow evolution from PC, inter-lane dependency, and low illumination inside the tunnel, and the “black hole” and “white hole” effects, respectively. If PC occurs at the rear location of serial tunnels, the density of high SC risk on the PC occurrence lane is relatively low since drivers maintain a slower speed when traversing the serial tunnels. Periodic oscillations of the traffic flow pattern are presented near tunnel portals when traffic flow departs from the PC occurrence location after PC removal.

In serial tunnels, creating a good lighting condition for drivers is more effective than advanced warnings in CVs to mitigate the SC risk after PC occurrence. To be specific, ATLC can alleviate the SC risk at the tail of a PC-incurred queue when the tail reaches the portal areas through eliminating the “black hole” and “white hole” effects. ATLC can considerably reduce the impact of inter-lane dependency and low illumination on crash risk along the adjacent lane of PC occurrence. ASLG for CVs manages to better alleviate the SC risk at the tail of the PC-incurred queue via immediately informing drivers in CVs of the hazardous traffic flow evolution from PC. However, a few new SC risky locations appear which result from the advance deceleration of CVs whose drivers decide not to change to the adjacent lane. Additionally, ASLG fails to limit the crash risk along the adjacent lane of PC occurrence since it cannot eliminate the inter-lane dependency after it guides a part of CVs to the adjacent lane. Hence, countermeasure combining ATLC with ASLG is promising.

Further research directions can be identified from the limitations of this paper. More factors affecting driving behavior need to be considered in LMTID, such as the narrowness effect of tunnel wall and visual fatigue travelling through serial tunnels. More video records after PC occurs are needed to more sophisticatedly calibrate and verify the LMTID and SC risk.

## Figures and Tables

**Table 1:** Truncated normal distribution parameters of MADR.

Distribution Parameters for MADR	Car	Truck
$M_{n}^{\mu }$ (m/s^2^)	8.45	6.82
$M_{n}^{\sigma }$ (m/s^2^)	1.40	1.40
$M_{n}^{up}$ (m/s^2^)	12.68	10.05
$M_{n}^{low}$ (m/s^2^)	1.23	0.60

**Table 2:** Behavioral parameters of vehicle-following patterns in CF part of LMTID.

	CC	CT	TC	TT
${\tilde{h}}_{n}$ (s)	1.2	1.4	1.8	2.0
$s_{n}^{\mathrm{jam}}$ (m)	1.04	1.62	1.23	1.89
$v_{n}^{0}$ (km/h)	80	57	61	52
$a_{n}^{\max}$ (m/s^2^)	1.01	1.03	0.78	0.74
$a_{n}^{\mathrm{comf}}$ (m/s^2^)	2.26	2.12	1.70	1.61

**Figure A1:**
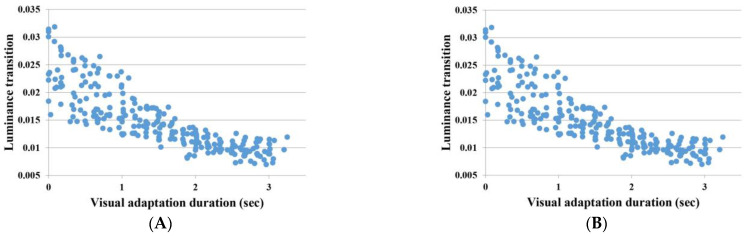
Relationship between LT and VA duration. (**A**) At entrance zone; (**B**) at exit zone.

**Figure A2:**
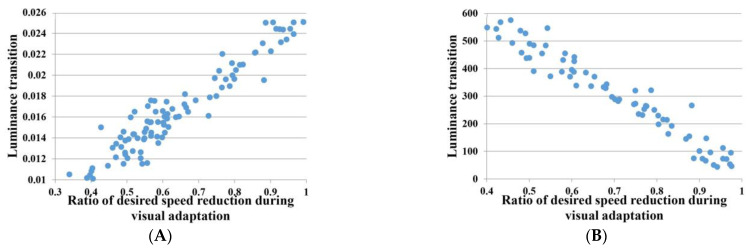
Relationship between LT and ratio of desired speed reduction during VA. (**A**) At entrance zone; (**B**) at exit zone.

**Figure A3:**
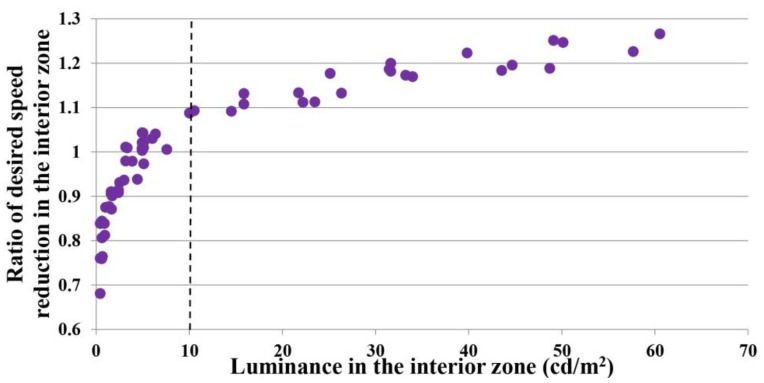
Relationship between luminance and ratio of desired speed reduction in the interior zone.

## Data Availability

The data presented in this study are available on request from the corresponding author.

## Appendix A. Calibration of Lighting-Related Behavioral Parameters

To evaluate the lighting-related behavioral parameters in LMTID, VA duration, and desired speeds in the tunnel, a total of 31 freeway tunnels in Chongqing, China were involved in the field test or driving simulator. The selected tunnels have different lighting conditions and the lengths varied from 1534 to 5392 m. In total, 50 drivers were recruited who were experienced drivers for at least 5 years and had fine visual ability. The tests were conducted from 9:00 a.m. to 5:00 p.m. on June of 2020, April of 2021, and April and May of 2022.

In this study, three experiments were conducted simultaneously during the course of driving in the field test: the relationship between the VA duration ($T_{a}$)/ratio of the desired speed reduction during VA ($b_{a}$) and LT ($L_{t}$) in the portal areas, the relationship between the ratio of desired speed reduction ($b_{in}$) and luminance ($L_{in}$) in the interior zone. In addition, a driving simulator experiment in the laboratory was supplemented to develop the relationship between $b_{in}$ and $L_{in}$.

On a sunny or cloudy day (totally 83 days), 50 participants were recruited in the field test where each of them drove through the 30 tunnels once in the morning and afternoon, respectively. The luminance is derived from the on-board illuminometer during the process of driving. The desired speed is obtained by GPS module, video recording module, and IMU. VA duration is measured by the oscillation of the driver’s pupil area, collected from portable eye-tracking system. Since the objective of the field tests is to evaluate the lighting-related behavioral parameters in the model, collected data are removed when the preceding car appears in the driver’s vision during the course of driving for all 50 drivers. The measurements of position, illuminance, car’s travel speed, and pupil area of drivers were tuned to ensure the consistency of the timeline of measurements.

### Appendix A.1. VA Duration and Desired Speed Reduction in Portal Areas

In the analysis, the average value for VA durations is used if the values of some LT are similar in different days or tunnels. The relationship between $L_{t}$ and $T_{a}$ is demonstrated in Figure A1. In Figure A1, each point (i.e., sample) is derived from a driver traversing a tunnel with its particular lighting condition. Points are presented only if VA occurs (i.e., VA duration is bigger than 0 s).

As Figure A1 indicates, the logarithm function is considered to be appropriate for quantifying the impact of LT on VA duration, as presented in Equation (A1).

(A1) $$T_{a}\left(L_{t}\left(x_{n}\left(t\right)\right)\right)=\left\{\begin{array}{ll} -2.976\ln L_{t}\left(x_{n}\left(t\right)\right)-11.188\left(s\right) & L_{t}\left(x_{n}\left(t\right)\right)\leq k_{1},R^{2}=0.78\\ 1.275\ln L_{t}\left(x_{n}\left(t\right)\right)-5.470\left(s\right) & L_{t}\left(x_{n}\left(t\right)\right)\geq k_{2},R^{2}=0.77 \end{array}\right.,\;$$

where R denotes the coefficient of determination reflecting the goodness of curve fit. In addition, the threshold for whether the VA occurs is determined as the intercept on the vertical coordinate of the logarithm function. The thresholds for dark and bright adaptations are $k_{1}=0.025$ and $k_{2}=73$, respectively.

In the desired speed reduction analysis, for each period (morning or afternoon) of a particular tunnel, the speed data of all 25 drivers is considered as a sample. The data of drivers are removed where VA does not occur. The relationship between $L_{t}$ and $b_{a}$ is demonstrated as Figure A2.

Linear function is appropriate to describe the impact of LT on the ratio of desired speed reduction, as presented in Equation (A2).

(A2) $$b_{a}\left(L_{t}\left(x_{n}\left(t\right)\right)\right)=\left\{\begin{array}{ll} 41.841L_{t}\left(x_{n}\left(t\right)\right)-0.059 & L_{t}\left(x_{n}\left(t\right)\right)\leq k_{1},R^{2}=0.93\\ -0.001L_{t}\left(x_{n}\left(t\right)\right)+1.100 & L_{t}\left(x_{n}\left(t\right)\right)\geq k_{2},R^{2}=0.96 \end{array}\right.$$

### Appendix A.2. Desired Speed Reduction in Interior Zone

The desired speed of a particular lighting condition in the interior zone is determined as the average value of collected speeds at all locations where no preceding vehicle appears in the visions of drivers. For each driver, the average value for desired speed is used for the analysis if the interior-zone lightings of different tunnels are close. The relationship between $L_{in}$ and $b_{in}$ is demonstrated in Figure A3.

Binary logarithm of $b_{in}$ has a linear relationship with the denary logarithm of $L_{in}$, and therefore we have

(A3) $$b_{in}\left(L_{in}\right)=1.818\times 2^{0.157\log L_{in}}-0.944,\;R^{2}=0.97$$
